# Old Eyes and Spectacles

**Published:** 1875-07

**Authors:** Swan M. Burnett

**Affiliations:** Knoxville, Tenn.


VOL. V.—JULY, 1875.—NO. 7.

### Original Communications.

### OLD EYES AND SPECTACLES.

BY SWAN M. BURNETT, M. D., KNOXVILLE, TENN.

It is in strict accordance with the laws governing the human economy, that there should be a failure in accurate vision with advancing years. Some facts, therefore, respecting the nature of this failure, and the proper means for remedying it, cannot be devoid of interest to all those to whom accurate vision is necessary.

Some knowledge, however, of the physiology of vision is necessary to a correct appreciation of the changes that occur in an eye that has become affected with age. As is well known, the eye possesses the power to accommodate itself for accurate vision at different distances, the objects in a landscape hundreds of yards away, and the letters of a book held twelve inches from the face, being all in a normal, healthy eye, seen with distinctness. But all are not seen with distinctness at the same time; if the eye is accommodated for the landscape it cannot make out the letters of the book, and *vice versa*. The reason of this is, that when the eye is accommodated for the objects in the distance, from which the rays of light proceed in a parrallel direction, the divergent rays coming from objects nearer at hand are not by the refracting surfaces of the eye brought to a focus on the retina so as to form a distinct image; and when on the other hand, we have distinct images of near objects, the parallel rays from a distance cannot have their focus on the retina. In other words, near and remote objects cannot have their images formed with clearness in the same plane without a change in the focal distance of the refracting surface. In the eye, this change is brought about by an alteration in the convexity of the crystalline lens, its convexity being increased for vision at near distances, and decreased for those more remote, until they attain the distance of twenty feet, at which point rays of light become practically parallel, and continue so up to an infinite distance. For viewing objects, then, beyond twenty feet, the normal eye is in a state of rest, but for accurate vision, at nearer distances, an effort must be put forth, to produce, by means of the ciliary muscle, the necessary increase in the convexity of the crystalline lens. It is evident that the nearer the object, the greater must be the increase in the curvature of the lens.

It is a diminution in this power of accommodation that constitutes *presbyopia* or old eyes. There are two factors operating to cause this loss. First, a stiffening of the ciliary muscle, and secondly a hardening of the substance of the lens, which prevents it from readily altering its shape. Of these, the latter is the most powerful, if indeed it is not the only factor, especially in the beginning and early course of, the trouble; for presbyopia begins, as a rule, before the general powers of the system begin to manifest any marked signs of failure; and it is hardly supposable that the ciliary muscle would begin to lose its activity so much earlier than the other parts of the muscular system.

This being the accepted cause and nature of presbyopia, it is not necessary to enter into a discussion of what it is not, or to consider the various erroneous opinions afloat concerning the producing causes of old eyes, such as flattening of the cornea of the lens, or of the whole eye-ball.

It is only in very recent times, that a distinction has been made between presbyopia and far-sightedness. There is a very essential difference in the two affections, for a knowledge of which we are indebted to the labors of Donders, Helmboltz, Knopp and others of the modern school of ophthalmologists. Far-sightedness is an abnormal, while presbyopia is a normal condition, and affects all eyes after they have attained a certain age. In far-sightedness, either the eye-ball is too short, or there is not a sufficiently strong refraction of the rays of light, for parallel rays, in a state of rest of the "accommodation," are not brought to a focus upon the retina, but behind it. Short-sightedness is the opposite condition to far-sightedness, and not to presbyopia as was once held, for myopic eyes themselves become presbyopic in time. In short, it may be said that while far-sightedness and near-sightedness are errors in the refraction of the eye, the former having too little, and the latter too much refraction, presbyopia is an affection of the accommodation, in which its power is diminished.

Though we denominate the diminution of accommodative power "old eyes," the changes on which this loss depends commences very early in life, before puberty, in fact. According to the calculations of Donders, the loss for every four or five years is represented by a lens of twenty-four inches positive focal length. Prof. H. Knopp, of New York, has found the annual loss to be represented by a similar lens of two hundred inches focus. There are, of course, individual exceptions to this rule, but it has been established as a working law, that persons of the same age, other things being equal, require glasses of about the same strength.

If, then, the changes which produce the condition of presbyopia take place so gradually, the time when eyes are to be considered old must be purely arbitrary and conventional. This has been fixed by Donders, and accepted generally by oculists, as the time when the nearest point of distinct vision with both eyes has receded to eight inches. Any eyes, therefore, which cannot read the ordinary newspaper print with facility at a distance of eight inches, are to be considered presbyopic.

As myopes use very little of their accommodative power, of course they will not feel the loss of it until that loss has been considerable; and, if the degree of myopia is at all high, say $\frac{1}{8}$, the loss of the whole of the accommodative power would not be felt.—It is a common observation that short-sighted persons in their old age are able to do without the assistance of glasses of any kind.—This is only, however, when the affection is of very low degree, for such is the nature of myopia that it shows a marked tendency to increase with age; and instead of diminishing the strength of his near-sighted glasses as he ascends in years, the myope has most commonly to increase them.

On the other hand, the far-sighted person will find that even though he may have previously used glasses that enabled him to read with ease at ten or twelve inches, he will have to increase the strength of his glasses about the thirtieth year or even sooner.

The ordinary signs of presbyopia are well known. When a book or newspaper is first taken up, the letters for an instant are indistinct, and it is instinctively removed further from the eye.—The foot-notes in finer print are passed over unread, and complaints frequently made against the bad illumination of the room. In the evening the printer is accused of carelessness in his work, the n's and u's, and 3's and 5's are confounded; and the reading is laid aside much sooner than formerly. Some months are generally passed in this way, when matters become worse instead of better; a friend some years older, hearing the complaints, suggests that glasses would set things all right and proffers her own for trial.—They are reluctantly taken, for few of us like to acknowledge this first indication that time has laid his inexorable finger upon us, but a glance through them generally at once proves the friend to be correct. Through his spectacles the print looks clearer and more distinct ; and the page can be brought much nearer to the eye and still be read with much ease.

It is plain, from what has gone before, that the object in making use of glasses is to make up, artificially, the loss in the power of accommodation ; the crystalline lens not having the power now to make itself sufficiently convex for accurate vision at near distances, the deficit must be made up by a lens placed exterior to the eye. It is also evident, that this lens must be neither too weak, nor too strong ; else, by deranging the balance of power of the ocular muscles, we shall cause as much discomfort and annoyance as we had before. A few plain rules will enable any intelligent person to fit his eyes comfortably with spectacles, though it is always best, where it is possible, to consult a competent oculist on the subject, since there may be some individual peculiarities in the case requiring a modification of the general law in the application of glasses. A very important question arises just here. When shall the use of glasses be begun ? There is a belief current among the laity, and even among some of the profession, that the use of glasses should be put off as long as possible, and that, by refraining from the use of glasses for as long a time as possible, the eyes are strengthened. This is a great error. Glasses should be applied just as soon as the loss of accommodative power calls for their aid. So soon as reading in the evening becomes irksome, and there is a manifest tendency to remove the reading away from the eye in order to render the letters more distinct, the aid of glasses should be sought. Instead of being an injury, it is a positive good to begin the use of spectacles as soon as the accommodation begins to fail. As long as one cannot see distinctly, there will be an instinctive effort to improve the vision, and this will result in a strain to the ocular muscles which must do harm where the balance of muscular power is so delicately poised as it is in the eye.

In normal healthy eyes, the need of some artificial aids to vision is commonly felt about the 45th year, and glasses will be found to give much comfort in reading or close work, especially in the evening and on dark days. Generally, the glasses needed in such cases is one of sixty inches focus. After the lapse of some time these glasses will be found necessary for constant use in daylight, and a stronger pair may be used at night. The ratio of increase of glasses is pretty constant in eyes that are healthy; and a table has been constructed, showing the number of the glass that corresponds to a particular year. The following is such a table:

| Age. | 48 | 50 | 55 | 58 | 60 | 62 | 65 | 70 | 75 | 78 | 80 |
|---|---|---|---|---|---|---|---|---|---|---|---|
| No. of Glass. | 60 | 40 | 30 | 22 | 18 | 14 | 13 | 10 | 9 | 8 | 7 |

This table, while it does not propose to be strictly accurate, approaches very nearly to accuracy in eyes that are not short or long-sighted, and are not otherwise diseased. If glasses stronger are needed than are called for at their corresponding ages in the table, the probabilities are that the individual is hyperopic, or far-sighted; and, if the glasses have to be changed very rapidly—two or three times a year for instance—and especially if this is connected with periodical "muddiness" of vision and more or less pain around the eye, the suspicion is strong in favor of that very insidious and destructive disease, *glancoma*, and the advice of a competent medical man should at once be sought.

It is needless to say, after what has already been said, that the same glasses cannot have accurate vision for both near and distant objects. If the eyes are at all of the hyperopic or far-sighted build—that is, too short—two pairs of glasses will generally be necessary: a weaker pair for distant vision and a stronger pair for near work. It does not by any means follow that the glasses should be changed so often as indicated in the table. These figures were calculated to show the very best vision at a certain fixed distance, say 18 inches from the eye; but, as the eyes become older, good vision is still retained by removing the object farther from the eyes; and, when this distance becomes so great as to be inconvenient, these glasses should be laid aside and those taken which at the corresponding age brings its point of most distinct vision to the required distance; that is about 18 inches.

The quality of glass from which the spectacles are made deserves notice. Some persons have a mania for "pebbles," and often enormous sums are paid for them; an impression seems to be abroad that vision through these glasses is much sharper than through others, and that they "strain" the eyes less. The pebble has one advantage, and only one: it is usually very hard, and therefore not easily scratched. A good, clear flint glass is, for all practical purposes, quite as good as the costilest pebble. For very strong lenses the crown glass is, perhaps, better than the flint, as its dispersive power is less; and in lenses of short focus the colored rings around objects would be annoying.

Those glasses that are to be used alone for distance are best of the perescopic form; but when they are to be used only for near work, the ordinary double-convex form is as good.

The spectacle frame is by no means an unimportant matter. Spring-clips over the nose, while they are convenient for momentary use, are not to be recommended for any prolonged use of the eyes. In using them the distance between the centres of the lenses is not always the same, and cannot always be made to coincide with the centres of the corneæ, as they should. If the centres of the glasses lie farther apart than the visual axes, when the latter are parallel, they will act as prisms with their bases outward; and, if they are nearer together than the visual axes, they perform the part of prisms with their bases inward. This would destroy the balance of power in the ocular muscles, and in some cases give rise to no little discomfort in the use of glasses. Sometimes it is advantageous to call this prism-action of the spherical glasses into requisition, in order to accommodate a weakened muscle; but in such cases the advice of a qualified oculist should *always* be sought. In order to bring the centres of the cornea and those of the spectacles into the same line, it is only necessary to get a friend to mark on a piece of paper held across the root of the nose, the centres of the pupil whilst looking at a distance, or to measure it one's self by looking at a mirror. We have only then to make the distance from the *outer* rim of one glass to the *inner* rim of the other, equal to the distance between the centres of the pupils, to have a coincidence between the axes of the vision for distance and the centres of the glasses, if the latter have been correctly ground. The distance from the eyes to the glasses should be about half an inch.

The best material for cleansing the glasses is a soft cotton cloth. Both linen and silk, as well as paper, are liable to scratch the glass. Soft buckskin and chamois leather are good, but are apt to carry grit.

